# Implementing a new tuberculosis surveillance system in Zhejiang, Jilin and Ningxia: improvements, challenges and implications for China’s National Health Information System

**DOI:** 10.1186/s40249-021-00811-w

**Published:** 2021-03-10

**Authors:** Wei-Xi Jiang, Fei Huang, Sheng-Lan Tang, Ni Wang, Xin Du, Hui Zhang, Yan-Lin Zhao

**Affiliations:** 1 Global Health Research Center, Duke Kunshan University, No. 8 Duke Avenue, Kunshan, 215316 Jiangsu China; 2 National Center for Tuberculosis Control and Prevention, China CDC, No.155 Changbai Road, Changping District, Beijing, 102206 China; 3 Duke Global Health Institute, Duke University, 310 Trent Drive, Durham, NC 27710 USA; 4 National Center for Tuberculosis Control and Prevention, China CDC, No.27 Nanwei Road, Xicheng District, Beijing, 100050 China

**Keywords:** Disease surveillance system, Tuberculosis, Implementation science

## Abstract

**Background:**

China is still faced with the public health challenge of tuberculosis (TB), and a robust surveillance system is critical for developing evidence-based TB control policies. The Tuberculosis Information Management System (TBIMS), an independent system launched in 2005, has encountered several challenges in meeting the current needs of TB control. The Chinese government also planned to establish the National Health Information System (NHIS) aggregating data in different areas. The China National Health Commission-Gates TB Project Phase III launched a new TB surveillance system to address these challenges and also as a pilot for the countrywide implementation of the NHIS. This commentary highlights the improvements and challenges in implementing the new TB system and also discusses the implications for the roll-out of the NHIS.

**Main text:**

The new TB surveillance system piloted in each prefecture of the project provinces was designed based on the local information system under the unified principle of organizing patient information under a unique ID and realizing the function of data exchange. Upon mid-2019, the data exchange successful rate reached almost 100%, and the system showed good performance in data completeness. Major improvements of the new system included achieving automatic data extraction instead of manual entry, assisting clinical service provision, and the augmented statistical functions. The major challenges in the implementation and scale-up of the new system were the licensing issue and the diversities of infrastructures that hinder the promotion of the new system at a low cost. This pilot also accumulated experiences for the roll-out of the NHIS regarding the technical solutions of reforming current information systems as well as effective training approaches for the developers and users of the new system.

**Conclusions:**

The successful implementation of the new TB surveillance system in the three TB designated medical institutions demonstrated how the diverse infrastructures of the information system could be reformed to achieve the functions of automatic data extraction and data exchange and better cater to the needs of healthcare workers. This pilot also accumulated rich experiences and lessons learnt for developing technical solutions and personnel training for the scale-up of the NHIS.
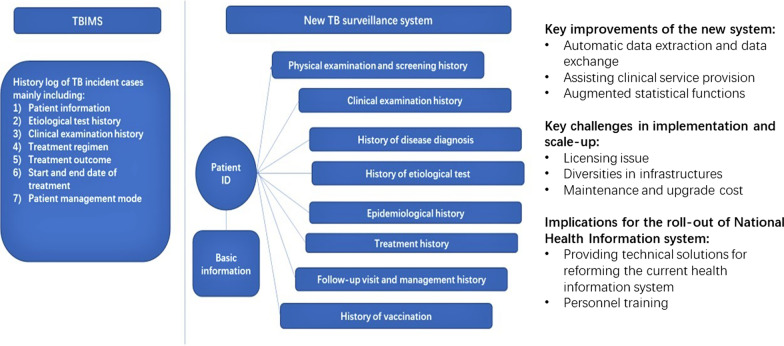

## Background

Despite impressive gains in recent decades, tuberculosis (TB) remains a public health challenge in China. China accounts for 8.4% of the global cases in 2019 according to the World Health Organization [1]. To achieve the Sustainable Development Goal of ending TB by 2030, developing a robust surveillance system with comprehensive and timely information is crucial for making evidence-based policy decisions [2]. In 2005, China established the Tuberculosis Information Management System (TBIMS), a web-based reporting and recording system for TB surveillance. Initially, TBIMS was rolled out across all TB health facilities, most of which were TB dispensaries affiliated with the center for disease control and prevention (CDC) [3]. TB dispensaries are required to enter all patients’ demographic, diagnostic, and treatment information within two days after diagnosis, and continuously update all test and treatment information during the treatment period. The data collected from the TBIMS has been used to inform China’s disease burden estimates and monitor the progress towards the implementation of the National Tuberculosis Program.

In recent years, TBIMS has encountered several challenges in meeting the current needs of TB control in China. China’s TB control model has been transitioning from the “CDC-led TB dispensary model” to an “integrated model”. This change shifted the location of TB service delivery away from the specialized TB dispensaries to the TB designated hospitals (i.e., infectious disease hospitals at city/prefecture level and beyond, or general hospitals at county/district level) with the goal of improving treatment quality [4]. Therefore, the responsibility of recording and managing data in TBIMS has been transferred to those physicians or nurses in the TB unit of the designated hospitals who already have a heavy workload. Furthermore, as TBIMS was designed mainly for disease surveillance, it could hardly assist physicians in providing clinical services, except more workload for them. Besides, the quality of data in the TBIMS may be undermined, as errors may occur in manual data entry. Underreporting of TB cases has been reported in previous studies both in China and other countries using similar electronic register systems, which could lead to an underestimation of TB disease burden in China and beyond [5–9]. Health workers may also “manipulate” some the records. For example, as physicians need to manage the records during the whole treatment period, they may just put a note to indicate treatment completion in the treatment records right at the standard treatment length to reduce the workload despite of the successive treatment, as revealed in the review of the registration data and previous studies on TB health service use [10]. Unintentional mistakes are also likely to happen during the manual entry procedure.

In recent years, there is a growing trend to establish a web-based platform aggregating real-time data for higher efficiency in infectious disease surveillance and control along with the fast development of information technology. Besides, the evidence-based policy making also requires more comprehensive information from different areas of healthcare. TBIMS has been running as an independent surveillance system since its establishment. Its current data structure and data transmission mode do not enable its integration into such a platform, and the data collected are not sufficient to inform comprehensive policy development. In 2016, China’s National Development and Reform Commission issued the *National Health Security Project Construction Plan*, which schematized the establishment of a National Health Information System (NHIS) that requires data aggregation from the six major areas of health including public health, health insurance, drug management, etc. through automatic data exchange [11]. The NHIS will serve as a comprehensive information platform to facilitate both healthcare seeking for patients as well as cross-sectoral healthcare management in terms of health resource allocation, healthcare cost containment, disease control, etc. The outbreak of the COVID-19 also highlights the urgency for more powerful and effective infectious disease surveillance systems with sufficient and timely information to support rapid disease control policy development at the early stages of the pandemic. In light of these challenges and the needs, the China National Health Commission (NHC)-Gates TB Project Phase III began to launch a new TB surveillance system in the TB designated medical institution of one prefecture in each of the three project provinces: Zhejiang, Jilin and Ningxia Hui Autonomous (referred as Ningxia) in 2016. The implementation of the new system also served as a pilot for the countrywide roll-out of the NHIS. The new TB surveillance system is structured similarly to the NHIS, and could be integrated as part of the NHIS after the implementation of NHIS.

This commentary examines the performance of the new TB surveillance system. Specifically, we highlight its improvements compared to TBIMS, summarize the experiences and examine the challenges in implementation and scale-up, and outline implications for the countrywide roll-out of the NHIS identified during the final project evaluation.

## Main text

### Response to challenges: implementing a new TB surveillance system

**Fig. 1:**
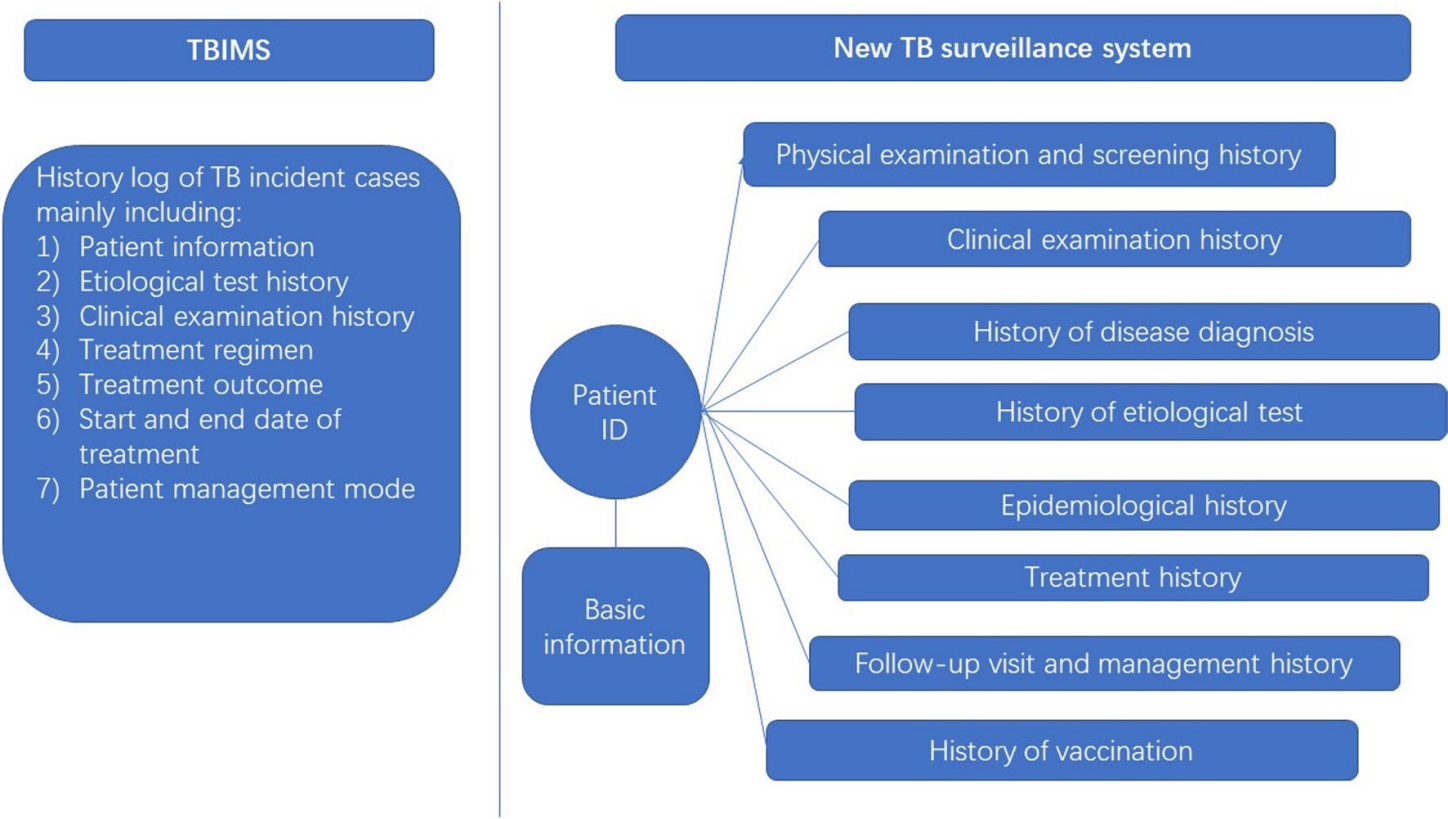
Structure of the information collected in Tuberculosis Information Management System (TBIMS) and the new TB surveillance system

The primary goal of the new TB surveillance system is to establish a basic surveillance database using patient ID as the primary key, and achieve the function of automatic data exchange between local hospital information systems and health information platforms at different administrative levels. As shown in Fig. 1, while data in the old system are organized as a simple history log for each TB incident case, the new system keeps all patient information under a unique patient ID, thus resolving the duplication problem. The new system also contains more comprehensive information than the TBIMS, such as detailed treatment records. This whole set of information of a particular patient can be easily accessed and clearly presented through searching the patient ID. The statistics functions of the system have also been improved in order to align with the needs of health workers.

The implementation of the new TB surveillance system was under the leadership of the National Project Management Office, and each project province developed their own pilot protocol with adaptation to local conditions while following the basic guiding principles. The basic standardized dataset was determined at the national level, and each province could add, but not reduce, the indicators required to be collected according to their own needs. As the three project provinces vary in the TB control model and the development level of information technology in the designated medical institutions, the design, implementation and the scale-up progress of the new system differ across provinces. In Zhejiang and Ningxia, the designated hospital refined its existing electronic medical record (EMR) system to include the modules of TB information collection, data extraction and exchange. In Jilin, TB patients could still receive treatment in TB dispensaries without EMR, therefore a new electronic TB information system designed according to the requirements of the national dataset was implemented in the TB dispensary involved in the pilot. The data exchange channels between the central and local platforms were tested multiple times using virtual data before uploading actual data to ensure the safety of data exchange. As for the scale-up, since the pilot hospital in Ningxia was the provincial-level designated hospital and also the only TB dispensary retained responsible for managing MDR-TB related health services in the province, the new information system has also been promoted to other prefecture- and county-level designated hospitals of Ningxia under the leadership of provincial-level hospital. In Zhejiang and Jilin, the new system has not been scaled up in other prefectures at the time of final project evaluation.

### Improvements of the new system and enabling factors for implementation

The evaluation team of China NHC-Gates TB Phase III conducted a comprehensive evaluation on the implementation of the new TB surveillance system through a mixed-method approach combining quantitative and qualitative research. The quantitative data were retrieved from the national platform of the new TB surveillance system and the old TBIMS system (still under operation during the pilot) to monitor the performance of the new system. Key informant interviews were conducted with the managers and developers, and focus group discussions were conducted with the users of the new system at the process evaluation in mid-2018, and the final evaluation in mid-2019. Results of the evaluation showed that the new system has been successfully implemented in the three pilot designated medical institutions. Quantitative results proved the completeness and timeliness of the data in the new TB surveillance system that the data exchanged from the three pilot institutions met the unified requirements of the national TB dataset, and the successful rate of data exchange were all close to 100% in mid-2019. The new system also showed good performance regarding the completeness of case records [12].

Qualitative research revealed several major improvements of the new system compared to TBIMS. First of all, the TB-related information could be automatically extracted and uploaded to the administrative platform timely when physicians enter patients’ information in the EMR, which avoided the additional burden of manual entry into another system and improved the data quality. In Zhejiang with relatively advanced information technology, the TB test equipment could be directly linked to the information system, thus the test results could also be automatically imported into the system without manual entry. Besides, as the new system contained much more information on patients’ clinical records that could be conveniently accessed using the patient ID, it also assisted physicians in understanding the treatment progress and adjusting treatment plan accordingly. Moreover, the statistics function included the calculation of more indicators and was also more user-friendly in terms of producing disease surveillance report. The logic check of data and the ID verification function embedded in the program also helped improve the data quality. In sum, the new TB information system collected more comprehensive and accurate data in a timely manner, while reducing physicians’ workload and catering to their needs in providing healthcare services.

Qualitative results also identified several key enabling factors that have facilitated the successful pilot of the new TB surveillance system. First is the strong leadership from the hospital director in terms of funding and coordination. The project funds from the sponsor were not enough to cover the cost of developing and implementing the new system, thus these hospitals needed to raise additional internal funds. Besides, as some healthcare workers were not willing to use the new system due to the errors at the test stage, it was crucial for the hospital director in charge to organize training workshops and let all users fully understand the improvements and advantages the new system could achieve in the near future. Moreover, establishing an effective communication channel between the developers and users is also critical for promoting the implementation of the new system. The instant response from the developers once receiving feedbacks from physicians or nurses, and the constant refinement of the functions would improve users’ confidence in the new system and better cater into their needs in providing clinical and public health related services.

### Challenges in the implementation and scale-up of the new system

There are many challenges in the implementation of such a new system in these project prefectures/cities. Our interviews with managers and users of the new surveillance system revealed several key barriers and obstacles seen during the implementation and scale-up of the new system. First of all, the hospital EMR in different prefectures were often developed by different companies, especially in Zhejiang where there are many companies providing EMR systems. Therefore, the new system successfully implemented in the pilot hospital could not be scaled up across the province at a relatively low cost due to the licensing issue. In Ningxia, the new system has been promoted steadily in hospitals whose EMR system was developed by the same company as the pilot hospital, while the progress for other hospitals was slow. Besides, for less-developed regions, the current infrastructure in some hospitals could not meet the requirements of a new surveillance system. For example, the test machines could not be linked to the electronic information system, and physicians still have to enter the results manually. Moreover, though the start-up cost of the new system was mostly covered through the project funds, the hospital managers voiced concern about the costs associated with maintaining and updating the new system when the pilot project ends. For the less-developed regions, this additional cost is especially burdensome without additional government support, as the information departments in the medical institutions are often not competent to handle the problems of the new system. Another common problem during the implementation process was that the new system usually needs a year of testing and modification before it can run successfully. Physicians generally complained about the double-burden of testing the new system while still performing tasks of the old system. Therefore, strategies need to be considered to shorten this testing period and improve the acceptability of the new system. In general, these challenges require additional government efforts to address and strong leadership to push forward for solutions.

### Lessons for the countrywide roll-out of the NHIS

The implementation of the new TB surveillance system has accumulated good practices and lessons for the roll-out of the NHIS. We found it crucially important to provide technical solutions and personnel training in the early stage of the implementation. First of all, the database of the NHIS launched across China in January, 2020 is structured similarly to the new TB surveillance system in that basic patient information and medical records are organized under a unique patient ID. Their basic function is also the same in that they both enable data exchange between different platforms. During the implementation of the new TB surveillance system, the technical difficulties in establishing the database, setting up a safe network environment and achieving automatic data exchange have been resolved under different infrastructures across the project provinces, thus providing valuable experiences on reforming the current information system to facilitate the countrywide roll-out of the NHIS. Besides, the CDCs of different levels in our pilot regions also updated the overall information system to ensure a steady and fast data exchange channel, and opened a network portal for data monitoring and generating statistical reports. These technical upgrades in health-related agencies are also essential preparations for implementing the NHIS. Moreover, essential training to both TB staff and software engineer is critical, which could help to better understand how to revise and update the local health information system to meet the requirements for data extraction and exchange. Experiences on providing efficient and effective training to system developers, maintainers and users were also accumulated through the pilot.

## Conclusions

The successful implementation of the new TB surveillance system in the three TB designated medical institutions demonstrated how the diverse infrastructures of the current information system could be reformed to achieve the function of automatic data extraction and data exchange between different platforms and better cater to the needs of healthcare workers. This pilot has also provided valuable experiences and lessons in terms of solving technical problems and training personnel for the countrywide implementation of the NHIS due to the similar nature of this new TB surveillance system and the NHIS.

## Data Availability

The datasets generated and analyzed during the current study are not publicly available due to the regulations of China CDC. Readers of the article need to discuss with China CDC and obtain their permission before the release of the dataset.

## Abbreviations

TB: Tuberculosis
TBIMS: Tuberculosis Information Management System
CDC: Center for Disease Control
NHIS: National Health Information System
NHC: National Health Commission
EMR: Electronic Medical Record
